# Using Artificial Intelligence to Develop a Multivariate Model with a Machine Learning Model to Predict Complications in Mexican Diabetic Patients without Arterial Hypertension (National Nested Case-Control Study): Metformin and Elevated Normal Blood Pressure Are Risk Factors, and Obesity Is Protective

**DOI:** 10.1155/2023/8898958

**Published:** 2023-02-16

**Authors:** Sergio A. Zaizar-Fregoso, Agustin Lara-Esqueda, Carlos M. Hernández-Suarez, Josuel Delgado-Enciso, Arturo Garcia-Nevares, Luis M. Canseco-Avila, Jose Guzman-Esquivel, Iram P. Rodriguez-Sanchez, Margarita L. Martinez-Fierro, Gabriel Ceja-Espiritu, Hector Ochoa-Díaz-Lopez, Francisco Espinoza-Gomez, Iyari Sanchez-Diaz, Ivan Delgado-Enciso

**Affiliations:** ^1^Facultad de Medicina, Universidad de Colima, Colima 28040, Mexico; ^2^Facultad de Psicología y Terapia de la Comunicación Humana de la Universidad Juárez del Estado Durango, Durango 81301, Mexico; ^3^Fundacion para la Etica Educacion e Investigacion del Cancer del Instituto Estatal de Cancerologia de Colima AC, Colima 28085, Mexico; ^4^Facultad de Ciencias Químicas Campus IV, Universidad Autónoma de Chiapas, Tapachula, 30700 Chiapas, Mexico; ^5^Instituto Mexicano del Seguro Social, Delegación Colima, Villa de Alvarez, 28983 Colima, Mexico; ^6^Facultad de Ciencias Biológicas, Universidad Autonoma de Nuevo Leon, San Nicolás de los Garza, 66455 Nuevo Leon, Mexico; ^7^Unidad de Medicina Humana y Ciencias de La Salud, Universidad Autonoma de Zacatecas, Zacatecas 98160, Mexico; ^8^Departamento de Salud, El Colegio de La Frontera Sur, San Cristóbal de Las Casas, 29290 Chiapas, Mexico; ^9^Subdirección de Prevención y Protección a la Salud, Instituto de Seguridad y Servicios Sociales de los Trabajadores del Estado, Ciudad de Mexico, 14070, Mexico; ^10^Instituto Estatal de Cancerología, Servicios de Salud del Estado de Colima, Colima 28085, Mexico

## Abstract

Diabetes mellitus is a disease with no cure that can cause complications and even death. Moreover, over time, it will lead to chronic complications. Predictive models have been used to identify people with a tendency to develop diabetes mellitus. At the same time, there is limited information regarding the chronic complications of patients with diabetes. Our study is aimed at creating a machine-learning model that will be able to identify the risk factors of a diabetic patient developing chronic complications such as amputations, myocardial infarction, stroke, nephropathy, and retinopathy. The design is a national nested case-control study with 63,776 patients and 215 predictors with four years of data. Using an XGBoost model, the prediction of chronic complications has an AUC of 84%, and the model has identified the risk factors for chronic complications in patients with diabetes. According to the analysis, the most crucial risk factors based on SHAP values (Shapley additive explanations) are continued management, metformin treatment, age between 68 and 104 years, nutrition consultation, and treatment adherence. But we highlight two exciting findings. The first is a reaffirmation that high blood pressure figures across patients with diabetes without hypertension become a significant risk factor at diastolic > 70 mmHg (OR: 1.095, 95% CI: 1.078-1.113) or systolic > 120 mmHg (OR: 1.147, 95% CI: 1.124-1.171). Furthermore, people with diabetes with a BMI > 32 (overall obesity) (OR: 0.816, 95% CI: 0.8-0.833) have a statistically significant protective factor, which the paradox of obesity may explain. In conclusion, the results we have obtained show that artificial intelligence is a powerful and feasible tool to use for this type of study. However, we suggest that more studies be conducted to verify and elaborate upon our findings.

## 1. Introduction

Diabetes mellitus (DM) is a metabolic disorder that causes abnormal blood glucose (BG) regulation, resulting in short and long-term health complications and even death if not properly managed. Unfortunately, there is no cure for DM [1, 2]. Hence, it is an increasingly prevalent chronic disease with patients prone to an increased morbidity and mortality rate [3, 4]. Mexico has a substantially high prevalence of metabolic disorders; 75.2% of the Mexican population is obese or overweight, 10.3% have DM, and 19.5% have dyslipidemia [5]. In addition, DM has been related to long-term neurological, microvascular, and macrovascular complications. Over time, it leads to neuropathy, nephropathy, retinopathy, and cardiovascular disease [6].

Complications from DM represent the leading cause of morbidity and mortality among the population with diabetes [7]. Therefore, generating information on risk factors for complications is highly relevant for developing tools that patients or physicians can use to anticipate the disease or its complications and take appropriate measures [8]. For example, the American Diabetes Association (ADA) diabetes risk test identifies the risk of developing DM2 [9], and the diabetic foot self-care questionnaire (DFSQ) helps to identify risks and prevent injuries or amputations of the feet [8].

Additionally, the use of prediction models to identify people at high risk of DM has been recommended by NICE [10], European guidelines for the prevention of type 2 DM [11], and the International Diabetes Federation [12]. Nevertheless, studies have yet to be conducted about DM, its complications, and risk factors [13].

Scientists can tailor learning models to find complex patterns within big data [14] using machine learning methods to effectively deliver integrative solutions for multiview data to explain an event or predict an outcome [15]. Furthermore, the ability of a model to find statistical patterns across millions of features and examples enables superhuman performance [16].

Due to the high prevalence of DM in the Mexican population and the lack of a model trained within such a population, we decided to develop a machine learning model on a national program's electronic medical records for patients with diabetes. Our goal is to identify risk factors for patients with diabetes complications through artificial intelligence.

## 2. Materials and Methods

### 2.1. Ethics

We have conducted the study under the Declaration of Helsinki and Guidelines on Good Clinical Practice. Furthermore, the ethics committee of the Faculty of Medicine of the University of Colima approved the project under the deidentification of medical records (2020-01-02). The protocol also complies with the FAIR principles for handling scientific data [17] and the three Mexican laws for managing and accessing confidential data of third parties [18–20]. Finally, we have followed the TRIPOD guidelines for the development and validation of predictive models [21].

### 2.2. Setting

We conducted a national nested case-control study [22, 23] with patients from 2011 to 2016 captured in Mexico's electronic medical record in the public health sector (ISSSTE) at the national level.

### 2.3. Participants

We have included all patients older than 18 years of age, diagnosed with diabetes mellitus type 2, and captured in the electronic medical record of the national program for patients with diabetes of the public health sector ISSSTE of Mexico, included in the period from 2011 to 2016. In addition, we have excluded all records from outside the period and all patients with hypertension patients. Finally, we have eliminated all empty and duplicated records.

### 2.4. Outcome

We considered five complications: amputations, myocardial infarction, stroke, nephropathy, and retinopathy. They have all been joined into a category labeled as a chronic complication.

### 2.5. Cases

Every patient within the study population with a physician's diagnosis of one or more of the five events of interest in their medical record.

### 2.6. Controls

Every patient within the study population with no physician's diagnosis of any of the five events of interest in his medical record.

### 2.7. Predictors

Among the variables included, we noted information on addictions, obstetric-gynecological history, medications (antidiabetic, antihypertensive, and lipid-lowering), physical activity, acute complications (ketoacidosis, hyperglycemia, hyperosmolarity, and hypoglycemia), the performance of routine tests (blood, urine, eyes, and feet), education, marital status, infections, hospitalizations, symptoms, referral to specialty, among others.

### 2.8. Sample Size

The sample was nonprobabilistic, in accordance with the minimum of 10 observations for each variable [24–26]. All patients diagnosed with diabetes mellitus type 2 treated by the ISSSTE MIDE program from 2011 to 2016 were included with the selection criteria specified above.

### 2.9. Missing Data

We deleted medical records containing empty records, and “no imputation” technique was used.

### 2.10. Statistical Analysis

All predictors used in the model were introduced in numerical form. The categorical variables were managed with dummy encoding, while the continuous numerical variables were stratified by quantiles [27, 28]. XGBoost is a machine learning algorithm based on decision trees with cross-validation. The patient ratio for training is 80% and 20% for validation. For each iteration on the dataset, the algorithm subselects a group that will act as an evaluation group. Throughout the whole training-testing process, we used a 5-element cross-validation. The evaluation group calculated the area under the curve, sensitivity, and specificity [29].

Machine learning models are usually seen as a “black box.” It takes some features as input and produces some predictions as output, “Shapley additive explanations” is the acronym for Shapley values, which are widely used in cooperative game theory. Essentially, Shapley's value measures the contributions to the outcome of each variable separately within the dataset while maintaining that their sum equals the outcome [30–33], which allows for a better interpretation of the results given by a machine learning model.

In addition, we have provided the odds ratio with its confidence interval, which provides a proper measure of association in case-control studies. In probability models, odds ratios are used to compare the relative probabilities that the outcome of interest will occur given exposure to the variable in question [34, 35].

## 3. Results

### 3.1. Preprocessing Phase

The initial database consisted of 1,852,766 records and 166 columns (predictors), with 97,044 patients receiving medical care from 2011 to 2016. After preprocessing, such as normalization, elimination of outliers, elimination of columns of identity (empty), data type conversion, and application of inclusion, exclusion, and elimination criteria, the final database consists of 234,847 records and 215 columns with a total of 63,776 patients only diagnosed with DM.

The reduction in the number of patients is mainly due to excluding patients diagnosed with hypertension. The reason why the number of columns is increased in the final database is due to preprocessing. We used percentiles to categorize continuous numerical columns to study the influence of the strata on the variables of interest. Thus, in the end, we have 153 binary columns, 31 categorical columns, and 31 numeric variables.

### 3.2. Patient Characteristics

Men comprised 40.42% of the study population and women represented 59.58%. Of the patients, 10.69% suffered from alcoholism and 12.2% from smoking. There are 13 antidiabetic medications, 32 antihypertensive medications, and 8 lipid-lowering treatments. While 46.78% of patients took at least one antidiabetic drug, 14.71% took antihypertensive drugs, and 26.84% took lipid-lowering drugs. Concerning self-care, 50.98% were attached to treatment, 14.03% measured glucose by themselves, 68.79% received education on diabetes mellitus, and 64.90% performed physical activity. Only 2.95% had been hospitalized during the period studied, with only 1.80% referring to a specialty consultation. In addition, 15.63% had acute complications, while 51.66% had chronic complications. 23.40% presented at least one type of infection (oral, genital, skin, or feet). Preventive examinations are as follows: 14.42% of eyes, 50.09% of feet, and 43.57% of urine.

While the average age of the patients was 59.01 ± 10.75, the average diagnosis was 52.67 ± 9.63, and the time with DM was 14.42 ± 11.13. Anthropometrically, the population has a BMI of 30.64 ± 5.35, a weight of 76.82 ± 15.58, and a height of 1.6 ± 0.1. Systolic blood pressure is 129.73 ± 17.48, diastolic is 80.3 ± 9.9, and pulse pressure is 52.85 ± 15. The general lipid profile presents total cholesterol of 206.47 ± 52.19, HDL 46.85 ± 12.63, LDL 110.26 ± 36.57, and triglycerides 226.8 ± 118.06. Their glycemic control with HbA1c was 8.78 ± 2.26, and their fasting blood glucose was 249.61 ± 99.48.

For this study, we utilized 14 predictive models such as random forest, linear regression, support vector machines, and naive Bayes. We evaluated all of them with the same training and test data. In addition to having been assessed with the same metrics, we present them in the following table. Given these metrics, the best performant algorithm during the selection phase was the XGBoost model, which went into the thorough process of fine-tuning its hyperparameters. In Table 1, we show the results obtained by the XGBoost model to evaluate the model's final performance to predict the development of chronic complications in nonhypertensive diabetic Mexican patients after the pipeline of fine-tuning.

### 3.3. Top Predictors from the XGBoost Model for Chronic Complications

Table 2 presents the variables that the model determined to be 10 variables with the most significant predictive power for the development of chronic complications in Mexican patients with diabetes and without hypertension according to their SHAP value together with their odds ratio (OR), their confidence interval, and their *P* value.

The risk factors identified in descending SHAP order are continuing in management increases the odds by 1.75 times. Being treated with metformin increases the odds by 3.90 times. The age between 68 and 104 years increases the odds by 1.44 times. Having been referred and consulted about nutrition increases the odds by 1.66 times. Finally, attaching to the treatment increases the odds by 1.35 times (see Table 2).

### 3.4. In-Depth View of the Predictors for Chronic Complications

For a more detailed review of the results obtained from the model, refer to Table 3, which presents a breakdown of the total information competent to this model. The variables are grouped by topic for better cohesion and understanding of the results discussed below.

### 3.5. Additional Statistical Analysis

To complete the results section, we present two tables. In Table 4, where a Chi-squared test was performed for the binary variables, and Table 5, we performed the Mann–Whitney *U* test for the numeric variables. Although many articles on machine learning mention having performed these tests, it is not required to publish those results.

## 4. Discussion

Despite current scientific advances, the vast amount of data prevents humans from extracting the maximum benefit. This has become the main limitation for scientific research since as researchers, we cannot operate at the scope, scale, and speed necessary for the amount and complexity of the collected data. For this reason, artificial intelligence is the most appropriate tool to make the most of all this data and transform it into helpful information for doctors and patients [17].

For example, it was previously mentioned that the first studies consulted had preselected the predictive variables through a literature review and not a statistical method [36–39]. However, this study has allowed the exclusion and inclusion of variables to be performed through statistical discrimination that analyzes the machine learning model through the value of SHAP [30–33].

Despite this, there are authorities such as the Centers for Disease Control and Prevention (CDC) that issue publications with reported risk factors for patients with diabetes: smoking, overweight or obesity, physical inactivity, systemic arterial hypertension, and hyperglycemia [40, 41].

Starting with smoking, we agree that this risk factor increases the risk of suffering any of the five events studied by 2.045 times. However, our study dove deeper into smoking as a risk factor. Stratifying the patient's years of smoking, we find an almost constant risk ratio, where smoking from 1 to 56 years increases the risk 1.22-1.33 times.

Obesity has been recognized as a health risk factor for many years. The risk of morbidity and mortality associated with it is high, as is insulin resistance, which frequently causes diabetes. But a growing body of research indicates that patients with obesity have a higher chance of surviving [42–44]. According to the so-called “obesity paradox,” persons who are obese survive health issues more often than people who are average weight. People with obesity may have the metabolic reserves required to balance the increased catabolic load during a health issue. In our analysis (see Table 3), we have found that a BMI greater than 32 (obesity) is a protective factor, which aligns with the obesity paradox theory. A BMI of less than 30 is presented as a risk factor.

Turning to physical activity, we found that this variable acts as a risk factor with an increase of 1.33 times the probability of suffering from a chronic complication as a person with diabetes. We understand that these results are controversial. However, we remind readers that this study is based on what was recorded in the patient files. Therefore, there was no way physicians ensured that the patients that carried out the said activity knew the type of activity, its duration, or its intensity. Furthermore, given the study's observational nature, we limit ourselves only to reporting what was found, recognizing its limitations.

This study used arterial hypertension as an exclusion criterion for patients, so the hypertension effect cannot be reflected on the variables. However, we have the blood pressure data of the patients involved in this study. We found that diastolic pressure becomes a risk factor from 70 mmHg with an increase of 1.09-1.20 times the risk. On the other hand, systolic pressure is presented as a risk factor from 120 mmHg, increasing the risk 1.14-1.23 times. In addition, we calculated the pulse pressure, which was a risk factor from 40 mmHg with an increase of 1.04-1.20 times the risk. These results conclude that in patients with diabetes but without hypertension, high blood pressure does act as a risk factor even when there is no official diagnosis.

The JNC 8 recommends a therapeutic control goal in patients with diabetes and hypertension less than 140/90 mmHg without a clinical trial as evidence. Based on a national analysis of patients with nonhypertensive diabetics, we add evidence to this recommendation. Despite being a more stringent goal (120/70 mmHg), it seeks to prevent the same complications exposed by JNC 8 (mortality, myocardial infarction, and cerebrovascular events). We base this goal on the data presented in Table 3, which indicates that blood pressure in people with diabetes without hypertension is a significant protective factor at diastolic < 70 mmHg (OR: 0.84, 95% CI: 0.826, 0.853) or systolic < 120 mmHg (OR: 0.974, 95% CI: 0.957, 0.991).

After obesity, arterial hypertension is the second most common cause of cardiovascular disease, which is the leading cause of morbidity and mortality. Therefore, preventing, diagnosing, and controlling high blood pressure are a global health priority only achieved by measuring blood pressure. In today's modern world, self-measurement with validated devices would be beneficial to involve the patient in treatment and follow-up [45].

Thus, the CDC's last variable is the risk factor hyperglycemia. In this case, we find that hyperglycemia recorded as an acute complication is a risk factor that increases the probability of a chronic complication by 4.5 times. However, we can again use the stratification of the numerical variables to provide specificity to these variables. Thus, fasting blood glucose is presented as a risk factor from 185 mg/dL, increasing 1.16-1.7 times the probability of suffering from a chronic complication. In addition, the fasting glucose gives us a view of these patients' adaptation to the variation in their blood glucose. Studying it through HbA1c, it appears to be a risk factor from 6%, increasing 1.07-1.18 times the probability of a chronic complication.

In addition to the variables considered by the CDC, there are publications, such as Abhari et al. and James et al., mentioning that blood pressure variables should be studied independently with machine learning techniques [46, 47]. This was a reason we included the stratification of blood pressure and added pulse pressure.

Knuiman et al. reported that age is the primary variable related to complications of diabetes mellitus [38]. However, we believe that, in this case, we can provide specificity. Although the variable may be a risk factor, we found that age becomes a risk factor after 55 years, increasing the probability of a chronic complication by 1.01-1.44 times.

Ross et al. published that by including all the variables of the electronic medical record, there was a decrease in its predictive performance [48]. However, this decision was recognized as a strength of the study, given that it reflected the reality of the patients [48–50]. Furthermore, we conducted the study without altering the database and without imputation of values—allowing the algorithm and accompanying techniques to study the discriminant variables for the model as recorded by physicians at the primary care level.

Additionally, in 2018, Dagliati et al. carried out a study with a similar approach to the present [51]. However, a bias was committed by not testing more than one machine learning algorithm. Moreover, before the study, they used logistic regression in a European population, which may reflect a different trend than a Hispanic population [51]. Therefore, for this case, we decided to carry out a fit test with 14 models of machine learning.

It is important to note that this study was conducted to identify risk factors for chronic complications, encompassing amputations, myocardial infarction, stroke, nephropathy, and retinopathy, without focusing on any particular affliction. Nevertheless, our results have generated information that supports future research aimed at the genesis of applicable instruments or strategies to benefit patients. In this sense, we must continue promoting the use of current and validated strategies and instruments for the care and prevention of complications in patients with DM, such as the determination of the degree of stiffness of the ankle, the use of the Foot Posture Index, or the use of the DFSQ, beneficial to prevent injuries or amputations of the pelvic limbs [8, 52].

We are no strangers to the wide variety of initiatives on machine learning focused on diabetes mellitus that have been launched since 2005 in the United States, Europe, Asia, and Australia. The Nordic Precision Medicine Initiative is the largest, formed in 2015, and intended to collect data from more than one million Nordic citizens [53]. Unfortunately, no publication in Mexico has included any initiative to make a comparable database. However, this project can provide a starting point for such an initiative. In addition, we demonstrate that Mexican programs already have value-rich data collection to extract insights for our Mexican patients.

### 4.1. Limitations

We recognize that all the subjects in this study share the same pathology, diabetes mellitus type 2, declared in the inclusion criteria. Therefore, the results cannot be generalized to all individuals, although the complications studied occur in other conditions.

Machine learning can be applied to medical records to develop robust risk models. However, our healthcare system is reluctant to entrust a machine with a human's task with mistakes. However, if we change the vision, this may sound acceptable. Machine learning is not here to replace any human job. It is here to help as a second opinion based on our population's thousands or even millions of records [54].

There is a dissociation between machine learning developers, regulatory bodies, health services researchers, doctors, and patients to generate new or more in-depth knowledge about the pathologies under study [39]. In addition, our project only covered patients served by one of the three public systems in the country; therefore, collection initiatives from the other systems would contribute significant value to this type of study.

### 4.2. Strengths

Most studies do not use longitudinal data, employ relatively few predictive variables with a median of 27, and rarely develop multicenter models [48]. In our case, the data is longitudinal, we start with 166 variables, and the data comes from all over the country.

Furthermore, this is the first Mexican national study that uses machine learning to predict complications in Mexican patients with diabetes mellitus. The published articles have indicated their scale, volume, and quantity of medical records as strengths [55]. However, this study is more extensive in scale, volume, and with respect to patient records because a national program is used as a data source.

Therefore, we can refine their results and compare them with ours.

This work intentionally ignored factors such as the researcher's experience, the use of specific algorithms in the literature, dependencies of hypothetical variables, and the interpretability of the model [56]. Thus, favoring techniques such as recursive elimination of variables, cross-validation, and hyperparameterization improve performance without altering patient data.

In addition, we have used a hypothesis-free machine learning approach for the research question. Thus, a prediction model capable of detecting variables that had not been previously reported as risk factors for complications in this population of patients was created [57]. In some cases, this model resulted in the ability to specify the ranges in which a variable becomes a risk factor.

As far as we know, we have tried to extract as much information as possible from the data source and provide some new insights on the risk factors facing our Mexican population with diabetes mellitus.

### 4.3. Collecting

Although this section is separate from the structure, we included it because we know that we exposed a large amount of information, which was reflected in the tables and could not be discussed in the same way.

We remind readers that this study is not intended to find causes of effects but possible cofactors that lead to the effect. Therefore, we usually are brief in discussions to avoid misinterpreting the data. For this reason, we provide the tables with raw information as we obtained it after processing.

Many results may lack published support, but one of the study's objectives was to show the tool's scope with a certain degree of supervision without causing bias. We avoid theorizing or arguing through logic or experience that specific values do not adhere to what is published.

However, these contrasting results also support our starting premise. Each population provides different information. Perhaps the white European and North American people differ at a specific level from the Mexican population. Nevertheless, if we see it in general, the results are similar. Therefore, this study invites further studies with the Mexican people with artificial intelligence to provide our medical colleagues with accurate information about our patient population.

## 5. Conclusions

Artificial intelligence identified risk factors for complications in patients with diabetes with an AUC above 80%. Artificial intelligence provides a vision of medical data that is not influenced by the medical literature when utilized in exploratory medical data analysis. Various outcomes may result from this, such as new research, theories, or public health measures. The advent of personalized medicine appears increasingly likely given the large amount of data that can be exploited by this tool and the fact that the results are not all unexpected.

## Figures and Tables

**Table 1:** Evaluation metrics for the model of chronic complications in patients with diabetes.

Estimator	Value	Estimator	Value	Estimator	Value	CM	1	0
ACC	75.81	AUC	84.10	MCC	0.51	1	27238	9009
PR	73.66	RC	75.15	F1	74.40	0	9739	31514

ACC: accuracy; AUC: area under the curve; MCC: Matthew's correlation coefficient; PR: specificity; RC: sensitivity; *F*1: *F* score; CM: confusion matrix.

**Table 2:** Summary of the ten most important predictors for the model of chronic complications in patients with diabetes.

Predictor	Value^∗^	SHAP	OR	CI	*P*
Years smoking	(30.0, 38.0]	0.269083	0.388	[0.38, 0.396]	<0.001
Continues in the MIDE program	Yes	0.202862	1.756	[1.725, 1.787]	<0.001
Metformin	Yes	0.184438	3.908	[3.792, 4.029]	<0.001
Age	(31.999, 50.0]	0.134289	0.641	[0.629, 0.654]	<0.001
Maximum weight	(97.0, 116.5]	0.124975	0.560	[0.55, 0.57]	<0.001
Age	(67.0, 104.0]	0.085687	1.447	[1.417, 1.477]	<0.001
Nutrition consultation	Yes	0.079658	1.662	[1.626, 1.699]	<0.001
HbA1c	(2.999, 6.0]	0.075796	0.719	[0.705, 0.734]	<0.001
Deliveries	(3.0, 4.0]	0.069664	0.729	[0.717, 0.741]	<0.001
Attached to treatment	Yes	0.062306	1.351	[1.323, 1.379]	<0.001

OR: odds ratio; CI: 95% confidence interval; *P*: the value of statistical significance. This table presents the results obtained by the XGBoost algorithm when trained to predict the dependent variable of chronic complication. The order of the variables is descending according to their importance value (SHAP) to predict said variable. The value column presents the value used for each intervening variable during the training. In addition, the odds ratio (OR) values, confidence interval (CI), and statistical significance value (*P*) are presented. ^∗^The notation (#, #] represents a range that does not include the first value and includes the second value. For calculating odds ratios, values outside this range are used as the reference value, such as lower or higher values in the database.

**Table 3:** In-depth analysis of the predictors for the model of chronic complications in patients with diabetes.

Predictor	Value^∗^	SHAP	OR	CI	*P*
Smoking	Yes	0	2.045	[1.951, 2.144]	<0.001
Years smoking	(0.999, 10.0]	0	1.225	[1.203, 1.248]	<0.001
Years smoking	(10.0, 20.0]	0	1.3	[1.274, 1.326]	<0.001
Years smoking	(20.0, 30.0]	0	1.309	[1.281, 1.338]	<0.001
Years smoking	(30.0, 38.0]	0.269083	0.388	[0.38, 0.396]	<0.001
Years smoking	(38.0, 56.0]	0	1.338	[1.305, 1.371]	<0.001
MIDE follow-up: discharge	Yes	0	1.434	[1.366, 1.505]	<0.001
MIDE follow-up: continue	Yes	0.202862	1.756	[1.725, 1.787]	<0.001
MIDE follow-up: death	Yes	0	3.51	[1.83, 6.731]	<0.001
MIDE follow-up: abandonment	Yes	0	1.613	[1.322, 1.967]	<0.001
MIDE follow-up: secondary care	Yes	0	3.587	[3.163, 4.067]	<0.001
Antidiabetic Tx: acarbose	Yes	0	3.214	[2.881, 3.585]	<0.001
Antidiabetic Tx: phenformin	Yes	0	1.463	[0.545, 3.929]	0.450
Antidiabetic Tx: glibenclamide	Yes	0	3.166	[3.061, 3.275]	<0.001
Antidiabetic Tx: glimepiride	Yes	0	5.225	[3.823, 7.14]	<0.001
Antidiabetic Tx: glipizide	Yes	0	2.988	[1.323, 6.746]	0.008
Antidiabetic Tx: metformin	Yes	0.184438	3.908	[3.792, 4.029]	<0.001
Antidiabetic Tx: pioglitazone	Yes	0	5.603	[4.817, 6.518]	<0.001
Antidiabetic Tx: rosiglitazone	Yes	0	3.076	[2.542, 3.723]	<0.001
Antidiabetic Tx: sitagliptin	Yes	0	11.055	[9.378, 13.033]	<0.001
Antidiabetic Tx: tolbutamide	Yes	0	3.152	[1.672, 5.944]	<0.001
Age	(31.999, 50.0]	0.134289	0.641	[0.629, 0.654]	<0.001
Age	(50.0, 55.0]	0	0.913	[0.894, 0.933]	<0.001
Age	(55.0, 60.0]	0	1.013	[0.992, 1.034]	0.220
Age	(60.0, 67.0]	0	1.214	[1.19, 1.239]	<0.001
Age	(67.0, 104.0]	0.085687	1.447	[1.417, 1.477]	<0.001
BMI	(19.99, 25.20]	0	1.151	[1.128, 1.175]	<0.001
BMI	(25.20, 27.54]	0	1.06	[1.039, 1.082]	<0.001
BMI	(27.54, 29.85]	0	1.022	[1.001, 1.043]	0.038
BMI	(29.86, 32.88]	0	0.981	[0.961, 1.001]	0.067
BMI	(32.88, 60.0]	0	0.816	[0.8, 0.833]	<0.001
Maximum weight	(45.99, 76.0]	0	1.23	[1.206, 1.255]	<0.001
Maximum weight	(76.0, 85.0]	0	1.252	[1.227, 1.277]	<0.001
Maximum weight	(85.0, 97.0]	0	1.306	[1.279, 1.332]	<0.001
Maximum weight	(97.0, 116.5]	0.124975	0.56	[0.55, 0.57]	<0.001
Maximum weight	(116.5, 175.0]	0	1.274	[1.231, 1.319]	<0.001
Nutrition consultation	Yes	0.079658	1.662	[1.626, 1.699]	<0.001
Fasting glucose	(70.999, 120.0]	0	0.676	[0.662, 0.689]	<0.001
Fasting glucose	(120.0, 146.0]	0	0.762	[0.746, 0.778]	<0.001
Fasting glucose	(146.0, 185.0]	0	0.986	[0.966, 1.006]	0.167
Fasting glucose	(185.0, 250.0]	0	1.169	[1.146, 1.193]	<0.001
Fasting glucose	(250.0, 500.0]	0.025681	1.702	[1.667, 1.737]	<0.001
HbA1c	(2.999, 6.0]	0.075796	0.719	[0.705, 0.734]	<0.001
HbA1c	(6.0, 6.9]	0	1.078	[1.057, 1.1]	<0.001
HbA1c	(6.9, 7.8]	0	0.988	[0.968, 1.009]	0.273
HbA1c	(7.8, 9.3]	0	1.107	[1.085, 1.13]	<0.001
HbA1c	(9.3, 16.0]	0	1.189	[1.165, 1.213]	<0.001
Abortions	(0.999, 2.0]	0	0.797	[0.774, 0.82]	<0.001
Abortions	(2.0, 4.0]	0	1.252	[1.217, 1.289]	<0.001
Cesarean section	(0.998, 2.0]	0	0.821	[0.801, 0.842]	<0.001
Cesarean section	(2.0, 3.0]	0	1.218	[1.189, 1.249]	<0.001
Pregnancies	(0.999, 3.0]	0	1.141	[1.121, 1.162]	<0.001
Pregnancies	(3.0, 4.0]	0.050018	0.725	[0.713, 0.737]	<0.001
Pregnancies	(4.0, 5.0]	0	1.181	[1.148, 1.214]	<0.001
Pregnancies	(5.0, 11.0]	0	1.261	[1.235, 1.287]	<0.001
Deliveries	(0.999, 3.0]	0	1.17	[1.151, 1.19]	<0.001
Deliveries	(3.0, 4.0]	0.069664	0.729	[0.717, 0.741]	<0.001
Deliveries	(4.0, 10.0]	0	1.245	[1.221, 1.269]	<0.001
Self-care: attached to the treatment	Yes	0.062306	1.351	[1.323, 1.379]	<0.001
Self-care: self-medication	Yes	0	2.58	[2.466, 2.7]	<0.001
Self-care: diabetes education	Yes	0	1.346	[1.318, 1.374]	<0.001
Self-care: physical activity	Yes	0.055163	1.331	[1.303, 1.36]	<0.001
Diastolic pressure	(39.999, 70.0]	0	0.84	[0.826, 0.853]	<0.001
Diastolic pressure	(70.0, 80.0]	0	1.095	[1.078, 1.113]	<0.001
Diastolic pressure	(80.0, 190.0]	0	1.204	[1.175, 1.233]	<0.001
Systolic pressure	(79.999, 110.0]	0	0.815	[0.8, 0.829]	<0.001
Systolic pressure	(110.0, 120.0]	0	0.974	[0.957, 0.991]	<0.001
Systolic pressure	(120.0, 130.0]	0	1.147	[1.124, 1.171]	<0.001
Systolic pressure	(130.0, 242.0]	0	1.23	[1.204, 1.257]	<0.001
Pulse pressure	(1, 40.0]	0	0.853	[0.839, 0.867]	<0.001
Pulse pressure	(40.0, 50.0]	0	1.041	[1.022, 1.06]	<0.001
Pulse pressure	(50.0, 170.0]	0	1.205	[1.181, 1.228]	<0.001
Ketoacidosis	Yes	0.002285	0.561	[0.489, 0.644]	<0.001
Hyperglycemia	Yes	0.044642	4.5	[4.259, 4.755]	<0.001
Hyperosmolar	Yes	0	3.612	[2.766, 4.717]	<0.001
Hypoglycemia	Yes	0	2.563	[2.227, 2.949]	<0.001

Tx: treatment; OR: odds ratio; CI: 95% confidence interval; *P*: the value of statistical significance. This table presents the results obtained by the XGBoost algorithm when trained to predict the dependent variable of chronic complication. The order of the variables is descending according to their importance value (SHAP) to predict said variable. The value column presents the value used for each intervening variable during the training. In addition, the odds ratio (OR) values, confidence interval (CI), and statistical significance value (*P*) are presented. ^∗^The notation (#, #] represents a range that does not include the first value but does include the second value. For calculating odds ratios, values outside of the range are used as the reference value, such as lower or higher values in the database.

**Table 4:** Chi-squared test for binary variables between complicated patients versus uncomplicated patients.

Predictor	Patients		*x* ^2^ test
	Complicated	Uncomplicated	*P*
Smoking	4956 (15.04%)	2823 (9.16%)	<0.001
Antidiabetic Tx: acarbose	1230 (3.73%)	439 (1.42%)	<0.001
Antidiabetic Tx: phenformin	9 (0.03%)	7 (0.02%)	0.907
Antidiabetic Tx: glibenclamide	12816 (38.90%)	5007 (16.24%)	<0.001
Antidiabetic Tx: glimepiride	220 (0.67%)	48 (0.16%)	<0.001
Antidiabetic Tx: glipizide	21 (0.06%)	8 (0.03%)	0.040
Antidiabetic Tx: metformin	18271 (55.45%)	6072 (19.70%)	<0.001
Antidiabetic Tx: pioglitazone	992 (3.01%)	203 (0.66%)	<0.001
Antidiabetic Tx: rosiglitazone	391 (1.19%)	145 (0.47%)	<0.001
Antidiabetic Tx: sitagliptin	1506 (4.57%)	157 (0.51%)	<0.001
Antidiabetic Tx: tolbutamide	36 (0.11%)	13 (0.04%)	0.004
Self-care: attached to the treatment	21706 (65.88%)	10808 (35.06%)	<0.001
Self-care: self-medication	6142 (18.64%)	2805 (9.10%)	<0.001
Self-care: diabetes education	23160 (70.29%)	20710 (67.18%)	<0.001
Self-care: physical activity	21791 (66.14%)	19597 (63.57%)	<0.001
Ketoacidosis	302 (0.92%)	611 (1.98%)	<0.001
Hyperglycemia	6200 (18.82%)	1640 (5.32%)	<0.001
Hyperosmolar	225 (0.68%)	71 (0.23%)	<0.001
Hypoglycemia	635 (1.93%)	283 (0.92%)	<0.001
MIDE follow-up: discharge	2774 (8.42%)	992 (3.22%)	<0.001
MIDE follow-up: continue	20625 (62.60%)	8472 (27.48%)	<0.001
MIDE follow-up: death	39 (0.12%)	9 (0.03%)	<0.001
MIDE follow-up: abandonment	257 (0.78%)	133 (0.43%)	<0.001
MIDE follow-up: secondary care	968 (2.94%)	178 (0.58%)	<0.001
Nutrition consultation	9565 (29.03%)	3082 (10.00%)	<0.001

Complicated: total number of patients and percentage in whom physicians registered one or more of the five events of interest in their medical record. Uncomplicated: total number of patients and percentage in whom physicians registered none of the five events of interest in their medical record.

**Table 5:** Mann–Whitney *U* test for numerical variables between complicated patients versus uncomplicated patients.

Predictor	Patients		UMW test
	Complicated	Uncomplicated	*P*
Abortions	2.0 (IQR 1.0-2.0)	2.0 (IQR 2.0-2.0)	<0.001
Cesarean section	1.0 (IQR 1.0-2.0)	1.0 (IQR 1.0-1.0)	<0.001
Pregnancies	4.0 (IQR 4.0-5.0)	4.0 (IQR 4.0-4.0)	<0.001
Deliveries	4.0 (IQR 3.0-4.0)	4.0 (IQR 4.0-4.0)	<0.001
BMI	30.04 (IQR 27.03-33.33)	29.85 (IQR 26.84-33.49)	0.004
Maximum weight	93.0 (IQR 82.0-110.0)	116.5 (IQR 103.0-116.5)	<0.001
Age	59.0 (IQR 51.0-66.0)	59.0 (IQR 52.0-66.0)	0.277
Diastolic pressure	80.0 (IQR 80.0-90.0)	80.0 (IQR 70.0-80.0)	<0.001
Systolic pressure	130.0 (IQR 120.0-140.0)	126.0 (IQR 120.0-140.0)	<0.001
Pulse pressure	50.0 (IQR 40.0-60.0)	50.0 (IQR 40.0-60.0)	<0.001
Fasting glucose	279.0 (IQR 200.0-350.0)	200.0 (IQR 140.0-280.0)	<0.001
HbA1c	8.8 (IQR 7.1-10.7)	8.0 (IQR 6.8-10.0)	<0.001
Years smoking	25.0 (IQR 12.0-38.0)	38.0 (IQR 30.0-38.0)	<0.001

Complicated: total number of patients and percentage in whom physicians registered one or more of the five events of interest in their medical record. Uncomplicated: total number of patients and percentage in whom physicians registered none of the five events of interest in their medical record.

## Data Availability

All relevant data appear in the present study. The datasets used and analyzed during the current study are available from the corresponding author upon reasonable request.

## References

[1] Woldaregay A. Z., Årsand E., Botsis T., Albers D., Mamykina L., Hartvigsen G. (2019). Data-driven blood glucose pattern classification and anomalies detection: machine-learning applications in type 1 diabetes. *Journal of Medical Internet Research*.

[2] Woldaregay A. Z., Årsand E., Walderhaug S. (2019). Data-driven modeling and prediction of blood glucose dynamics: machine learning applications in type 1 diabetes. *Artificial Intelligence in Medicine*.

[3] Lai H., Huang H., Keshavjee K., Guergachi A., Gao X. (2019). Predictive models for diabetes mellitus using machine learning techniques. *BMC Endocrine Disorders*.

[4] Ogurtsova K., da Rocha Fernandes J. D., Huang Y. (2017). IDF Diabetes Atlas: global estimates for the prevalence of diabetes for 2015 and 2040. *Diabetes Research and Clinical Practice*.

[5] Romero-Martínez M., Shamah-Levy T., Vielma-Orozco E. (2019). Encuesta Nacional de Salud y Nutrición 2018-19: metodología y perspectivas. *Salud Pública de México*.

[6] Vehí J., Contreras I., Oviedo S., Biagi L., Bertachi A. (2020). Prediction and prevention of hypoglycaemic events in type-1 diabetic patients using machine learning. *Health Informatics Journal*.

[7] Dietrich I., Braga G. A., de Melo F. G., da Costa Silva Silva A. C. (2017). The diabetic foot as a proxy for cardiovascular events and mortality review. *Current Atherosclerosis Reports*.

[8] Navarro-Flores E., Losa-Iglesias M. E., Becerro-de-Bengoa-Vallejo R. (2022). Repeatability and reliability of the diabetic foot self-care questionnaire in Arabic patients: a transcultural adaptation. *Journal of Tissue Viability*.

[9] Aldayel F. A., Belal M. A., Alsheikh A. M. (2021). The validity of the American Diabetes Association’s diabetes risk test in a Saudi Arabian population. *Cureus*.

[10] NICE (2017). *Type 2 diabetes: prevention in people at high risk public health guideline*.

[11] Engelgau M. M., Narayan K. M., Herman W. H. (2000). Screening for type 2 diabetes. *Diabetes Care*.

[12] Alberti K. G., Zimmet P., Shaw J. (2007). International Diabetes Federation: a consensus on type 2 diabetes prevention. *Diabetic Medicine*.

[13] Madmoli M., Dehcheshmeh Z. M., Rafi A., Kord Z., Mobarez F., Darabiyan P. (2019). The rate of some complications and risk factors of diabetes in diabetic patients: study on cases of 3218 diabetic patients. *Medical Science*.

[14] Ngiam K. Y., Khor I. W. (2019). Big data and machine learning algorithms for health-care delivery. *The Lancet Oncology*.

[15] Cirillo D., Valencia A. (2019). Big data analytics for personalized medicine. *Current Opinion in Biotechnology*.

[16] Rajkomar A., Dean J., Kohane I. (2019). Machine learning in medicine. *The New England Journal of Medicine*.

[17] Aalbersberg I. J. J., Appleton G., Axton M. (2016). The FAIR guiding principles for scientific data management and stewardship. *Scientific Data*.

[18] INAI (2015). *Ley Federal De Transparencia Y Acceso a La Información Pública*.

[19] De los Ángeles Rios Ruíz A. (2017). Visión global de la Ley General de Transparencia y Acceso a la Información Pública. *Revista de la Facultad de Derecho de México*.

[20] Cámara de Diputados (2017). *Ley General de Protección de Datos Personales en Posesión de Sujetos Obligados*.

[21] Collins G. S., Reitsma J. B., Altman D. G., Moons K. G. M. (2015). Transparent Reporting of a Multivariable Prediction Model for Individual Prognosis or Diagnosis (TRIPOD): the TRIPOD Statement. *Diabetic Medicine*.

[22] Kleinbaum D. G., Calles D. L., Sullivan K. M. (2013). *ActivEpi Español*.

[23] Lazcano-Ponce E., Salazar-Martínez E., Hernández-Avila M. (2001). Estudios epidemiológicos de casos y controles. Fundamento teórico, variantes y aplicaciones. *Salud Pública México*.

[24] Demidenko E. (2008). Sample size and optimal design for logistic regression with binary interaction. *Statistics in Medicine*.

[25] Grieve A. P., Sarker S. J. (2016). Simulation-based sample-sizing and power calculations in logistic regression with partial prior information. *Pharmaceutical Statistics*.

[26] Vittinghoff E., McCulloch C. E. (2007). Relaxing the rule of ten events per variable in logistic and Cox regression. *American Journal of Epidemiology*.

[27] McKinney W. (2018). *Python for Data Analysis: Data Wrangling with Pandas, NumPy, and IPython*.

[28] Vanderplas J. T. (2016). *Python Data Science Handbook: Essential Tools for Working with Data*.

[29] Lee H., Lee E. J., Ham S. (2020). Machine learning approach to identify stroke within 4.5 hours. *Stroke*.

[30] Lundberg S. M., Erion G., Chen H. (2020). From local explanations to global understanding with explainable AI for trees. *Nature Machine Intelligence*.

[31] Parsa A. B., Movahedi A., Taghipour H., Derrible S., Mohammadian A. (. K.). (2020). Toward safer highways, application of XGBoost and SHAP for real-time accident detection and feature analysis. *Accident Analysis & Prevention*.

[32] Wu Z., Lei T., Shen C., Wang Z., Cao D., Hou T. (2019). ADMET evaluation in drug discovery. 19. Reliable prediction of human cytochrome P450 inhibition using artificial intelligence approaches. *Journal of Chemical Information and Modeling*.

[33] Messalas A., Kanellopoulos Y., Makris C. Model-agnostic interpretability with Shapley values.

[34] Szumilas M. (2010). Explaining odds ratios. *Journal of the Canadian Academy of Child and Adolescent Psychiatry*.

[35] Grimes D. A., Schulz K. F. (2008). Making sense of odds and odds ratios. *Obstetrics and Gynecology*.

[36] Wilson P. W. F. (2007). Prediction of incident diabetes mellitus in middle-aged adults. *Archives of Internal Medicine*.

[37] Cox D. R., Snell E. J. (1989). Analysis of Binary Data. http://search.ebscohost.com/login.aspx?direct=true&scope=site&db=nlebk&db=nlabk&AN=1838609.

[38] Knuiman M. W., Welborn T. A., McCann V. J., Stanton K. G., Constable I. J. (1986). Prevalence of diabetic complications in relation to risk factors. *Diabetes*.

[39] Broome D. T., Hilton C. B., Mehta N. (2020). Policy implications of artificial intelligence and machine learning in diabetes management. *Current Diabetes Reports*.

[40] CDC Risk Factors for Complications. https://www.cdc.gov/diabetes/data/statistics-report/risks-complications.html.

[41] Diabetes Care (2019). Summary of revisions: standards of medical care in diabetes—2019. *Diabetes Care*.

[42] Cheng C. C., Huang W. C., Chiou K. R. (2013). Body mass index and outcome of acute myocardial infarction-is there an obesity paradox?. *Acta Cardiologica Sinica*.

[43] Fonarow G. C., Srikanthan P., Costanzo M. R., Cintron G. B., Lopatin M., ADHERE Scientific Advisory Committee and Investigators (2007). An obesity paradox in acute heart failure: analysis of body mass index and inhospital mortality for 108 927 patients in the Acute Decompensated Heart Failure National Registry. *American Heart Journal*.

[44] Gao M., Sun J., Young N. (2016). Impact of body mass index on outcomes in cardiac surgery. *Journal of Cardiothoracic and Vascular Anesthesia*.

[45] Mazoteras-Pardo V., Gómez-Cantarino S., Ramírez-Jiménez M., Navarro-Flores E., Ugarte-Gurrutxaga M. I. (2023). Validations of blood pressure measuring devices using recognized protocols. *Journal of Personalized Medicine*.

[46] Abhari S., Niakan Kalhori S. R., Ebrahimi M., Hasannejadasl H., Garavand A. (2019). Artificial intelligence applications in type 2 diabetes mellitus care: focus on machine learning methods. *Healthcare Informatics Research*.

[47] James P. A., Oparil S., Carter B. L. (2014). 2014 evidence-based guideline for the management of high blood pressure in adults. *Journal of the American Medical Association*.

[48] Ross E. G., Jung K., Dudley J. T., Li L., Leeper N. J., Shah N. H. (2019). Predicting future cardiovascular events in patients with peripheral artery disease using electronic health record data. *Circulation. Cardiovascular Quality and Outcomes*.

[49] Géron A. (2019). *Hands-on Machine Learning with Scikit-Learn, Keras, and Tensor Flow: Concepts, Tools, and Techniques to Build Intelligent Systems*.

[50] Goodfellow I., Bengio Y., Courville A. (2016). *Deep Learning*.

[51] Dagliati A., Marini S., Sacchi L. (2018). Machine learning methods to predict diabetes complications. *Journal of Diabetes Science and Technology*.

[52] Simón-Pérez E., Simón-Pérez C., Alonso-Peña D. (2020). Stiffness degree of ankle range of motion in diabetic patients with atypical amputation. *Revista da Associação Médica Brasileira*.

[53] Klonoff D. C., Florez J. C., German M., Fleming A. (2020). The need for precision medicine to be applied to diabetes. *Journal of Diabetes Science and Technology*.

[54] Deo R. C. (2015). Machine learning in medicine. *Circulation*.

[55] Ayala Solares J. R., Canoy D., Raimondi F. E. D. (2019). Long-term exposure to elevated systolic blood pressure in predicting incident cardiovascular disease: evidence from large-scale routine electronic health records. *Journal of the American Heart Association*.

[56] Deist T. M., Dankers F. J. W. M., Valdes G. (2018). Machine learning algorithms for outcome prediction in (chemo)radiotherapy: an empirical comparison of classifiers. *Medical Physics*.

[57] Anderson J. P., Parikh J. R., Shenfeld D. K. (2016). Reverse engineering and evaluation of prediction models for progression to type 2 diabetes: an application of machine learning using electronic health records. *Journal of Diabetes Science and Technology*.

